# Neuron-based heredity and human evolution

**DOI:** 10.3389/fnins.2015.00209

**Published:** 2015-06-17

**Authors:** Don M. Gash, Andrew S. Deane

**Affiliations:** Department of Anatomy and Neurobiology, College of Medicine, University of KentuckyLexington, KY, USA

**Keywords:** human evolution, heredity, brain, mind, behaviorally-acquired information, cultural heredity, Neolamarckian

## Abstract

It is widely recognized that human evolution has been driven by two systems of heredity: one DNA-based and the other based on the transmission of behaviorally acquired information via nervous system functions. The genetic system is ancient, going back to the appearance of life on Earth. It is responsible for the evolutionary processes described by Darwin. By comparison, the nervous system is relatively newly minted and in its highest form, responsible for ideation and mind-to-mind transmission of information. Here the informational capabilities and functions of the two systems are compared. While employing quite different mechanisms for encoding, storing and transmission of information, both systems perform these generic hereditary functions. Three additional features of neuron-based heredity in humans are identified: the ability to transfer hereditary information to other members of their population, not just progeny; a selection process for the information being transferred; and a profoundly shorter time span for creation and dissemination of survival-enhancing information in a population. The mechanisms underlying neuron-based heredity involve hippocampal neurogenesis and memory and learning processes modifying and creating new neural assemblages changing brain structure and functions. A fundamental process in rewiring brain circuitry is through increased neural activity (use) strengthening and increasing the number of synaptic connections. Decreased activity in circuitry (disuse) leads to loss of synapses. Use and disuse modifying an organ to bring about new modes of living, habits and functions are processes in line with Neolamarckian concepts of evolution (Packard, 1901). Evidence is presented of bipartite evolutionary processes—Darwinian and Neolamarckian—driving human descent from a common ancestor shared with the great apes.

*I think that a new kind of replicator has recently emerged on this planet. It is staring us in the face. It is still in its infancy, still drifting clumsily about in its primeval soup, but already it is achieving evolutionary change at a rate that leaves the old gene panting far behind*.

Dawkins, 1976

*We regard the emergence of the nervous system as a major transition. The evolution of a nervous system not only changed the way that information was transmitted between cells and profoundly altered the nature of the individuals in which it was present, it also led to a new type of heredity – social and cultural heredity – based on the transmission of behaviourally acquired information*.

Jablonka and Lamb, 2006

## Introduction

The extraordinary development of human culture is one manifestation of highly developed cognitive capabilities characterizing anatomically modern humans. Here neuron-based heredity is analyzed as a principal component of hominin evolution leading to the current species with a highly developed capability for mind-to-mind exchange of information. The neuroplastic mechanisms involved, including the incorporation of new information into neural assemblages via neurogenesis in the hippocampus and learning and memory processes through strengthening (use) and weakening (disuse) of neural circuitry (Aimone et al., 2011; Anderson, 2011), serve the replicative functions posited by Dawkins. The heritage of acquired information provides the cornerstone for human culture.

As the cellular and molecular mechanisms of the two systems of heredity are very different, genetics and neurobiology have developed as separate scientific disciplines with often only modest overlap. The gene was rediscovered at the beginning of the twentieth century. Genetic processes provided the replicative biological mechanisms posited by Darwin in his theory of evolution. Support for Neolamarckism, the major competing theory, waned as its concepts were based on description, without evident testable biological mechanisms. Studies on genes and their role in heredity have flourished with exciting discovery after discovery capturing the attention of the public and scientists alike. The human genome has been aggressively sequenced along with that of other species. The science of genetics has matured to serve as a cornerstone of modern agriculture, biology, biotechnology and medicine.

In contrast, the second system of heredity has remained in the shadows. While the transmission of social and cultural information have long been fields of study in anthropology, the neurobiology of transmission of acquired information is still developing. Important studies of cultural evolution have added insights at the descriptive level, including emphasizing the role of non-genetic processes (Blackmore, 1999; Richerson and Boyd, 2005). But because of the difficulty in linking behavioral observations to neural processes, the nature of non-genetic contributions to bipartite human evolution has remained controversial (Boyd and Richerson, 2000; Kuper, 2000). However significant advances in understanding neuroplasticity and technical advances in neuroimaging and neurogenetics are now making it possible to correlate genetic factors and behavior with processes at the nervous system level.

## Limitations of gene-based heredity

One of the major surprises to come from mapping the human genome has been how few genes it contains. Traditionally, a gene has been defined as a DNA sequence encoding information for a specific protein. Using this criterion, the size of the human genome is humbling, with current evidence indicating it contains under 20,000 protein-coding genes (Ezkurdia et al., 2014). Plants such as rice and corn have many more genes than humans. Their genomes are in the 30,000+ gene range, more than 50% larger than the human genome (Goff et al., 2002; Schnable et al., 2009). The simple millimeter-long nematode worm *Caenorhabditis elegans*, which lives in the soil and feeds on bacteria, has 19,735 protein-coding genes, approximately the same number of as humans (Hillier et al., 2005).

With a life span under 4 weeks, the nervous system of an adult male *C. elegans* consists of 302 neurons (White et al., 1986). The human brain alone contains some 86 billion neurons (Herculano-Houzel, 2012), and the average human life span is more than 1000 times longer. While there can be increased complexity in the human genome, it is difficult to see how it can account for more than carrying a small fraction of the information needed for the development of the complex human brain with its large informational capacity. Indeed, the number, types and sequences of human genes are similar with those of other mammalian species with much smaller brains (Clamp et al., 2007).

## Mechanisms for encoding, storing, and transmission of information

Genetic information is encoded in nucleotide sequences and chromosomal structure of an individual's genome. Transcription and translation of encoded information are dynamic molecular processes regulating cellular life: responding to stimuli, maintaining homeostasis, and regulating growth, development and reproduction. There are various mechanisms for transmitting genetic information in single cells and multicellular organisms involving replication of the encoded information. In humans and many other species, sexual reproduction creates a unique combination of genes in a new transient single cell organism called a zygote combining genetic information from two individuals. The zygote rapidly develops into a multicellular organism with each daughter cell containing newly constituted genetic information from the zygote.

Genetic informational content is primarily determined at the time of conception. With some important exceptions such as mutations, epigenetic modifications and viral infections, genetic information is rigidly maintained in the germ cell line of the individual. Transmission of genetic information to the next generation occurs only with the fertilization of an ovum combining genetic material from two sexually competent individuals. Approximately 50% of genetic information from each parent is passed on to the offspring. The parents do not control the assortment.

Neuron-based informational content is accumulated and modified throughout life in the human nervous system. Information in the nervous system is encoded in the molecular and cellular properties of neurons, their neural networks and their synaptic connections. While the basic blueprint for organization and development of the nervous system is provided by an individual's genome, internal and external stimuli profoundly influence the development, structure and function of the nervous system. Informational content is generated and modified over the lifetime of an individual via experience, ideation, and additions, deletions and modifications of existing ideas. The mechanisms of action are those governing the elegant neuroplasticity of neurons, neuronal remodeling of structures and functions in response to incoming electrophysiological and chemical stimuli (Kandel, 2001).

The mechanism for transfer of neuron-based information from individual-to-individual in a population is via mind-to-mind. Mind-to-mind transfer engages the brain and body as well as the mind. As Damasio has emphasized, the mind in part can be conceived as a dynamic process between neural mappings of information received by exteroceptive sensory systems (see Table 1) referenced against interoceptive sensory input from the internal systems of the body (Damasio, 2010; Damasio and Carvalho, 2013). There is opportunity for transmission and reception of neuron-based information throughout an individual's lifetime. An individual can select the information being transferred (Taumoepeau and Ruffman, 2008; Heyes and Frith, 2014).

**Table 1:** **Mind-to-Mind Transfer of Information**.

**Individual A**				**Individual B**		
Brain	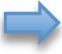	motor systems	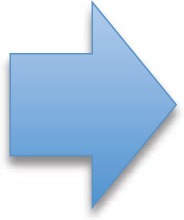	Sensory systems	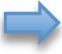	brain
Forebrain		Vocalization		Vision		Forebrain
Midbrain		Sign language		Hearing		Midbrain
hindbrain		Body language		Tactile		Hindbrain
Interoceptive sensory systems				Thermal Pain		Interoceptive sensory systems
				Olfaction		
				Taste		

*This simplified schematic diagrams the involvement of the body through interoceptive sensory neurons mapping the internal milieu providing moment-to-moment information to the brain for the mind's representation of “self” (Damasio, 2003). Consciously and non-consciously, the mind of one individual transmits information via vocalizations and body movements. In higher order communications, the body movements compose and use symbols (art, writing, etc.,) to transmit ideas. Exteroceptive sensory neurons in another individual receive the information and carry it to the brain where its interpretation is partially dependent upon the individual's memories and sense of self*.

As genes and neurons manage information by different mechanisms, the most meaningful comparisons between the two systems are seen in the functions they perform (Table 2). Many are generically similar. Both systems encode, store and transfer information; both receive internal and external information and generate responses. Both pass hereditary information on to descendants, although by quite different mechanisms. Both can generate new information leading to changes in populations that accumulate over time.

**Table 2:** **Human Gene-Based and Neuron-Based Heredity Systems**.

**Function**	**Genome**	**Nervous system**
(1) Manage encoded information	Yes, via DNA in genes and chromosomal organization	Yes, via neurons, synapses and neural networks
(2) Receive information (stimuli) from the local environment	Yes, via cell surface receptors.	Yes, via receptors of sensory neurons
(3) Respond to stimuli, transforming information into operant units	Yes, transcription of DNA to RNA, translation into proteins	Yes, neural networks process and store in-coming information, generating behavioral responses and ideation
(4) Generation of new hereditary information	Yes, via random mutations, sorting, resorting, additions and deletions of encoded information in genes and chromosomes	Yes, via experience, ideation, additions, deletions and modifications of existing ideas.
(5) Transmission Process	Sexual Reproduction	Mind-to-Mind
(6) Transmission of information to progeny	Yes, through genes	Yes, acquired information
(7) Transmission of information to other individuals in the population	No	Yes, acquired information
(8) Select the information being transmitted	No	Yes
(9) Time span for the creation and dissemination of new information enhancing survival	From generation to generation, i.e., decades to millennia	From seconds and minutes to years

However, there are three profound functional differences between the systems that have given rise to an increasingly powerful mind with extraordinary capabilities. Not only can the nervous system generate and pass on new information to progeny, it can also directly pass acquired information on to other members of the species. Next, the time span required for the nervous system to generate and disseminate new information is many orders of magnitude faster than the genetics system, as fast as in seconds for the brain compared to generational transfer (decades for humans) for the genome.

Finally, in contrast to genetic information passed on through sexual reproduction, individuals can choose some of the neuron-based information they transfer to others (Taumoepeau and Ruffman, 2008; Heyes and Frith, 2014). A prime example is the language and content mothers use in training their young children in culture-specific skills such as reading. Print reading and mind reading (interpreting/inferring other's states of mind, intentions and emotions) capabilities have genetic components as well as require learning acquired skills passed on from others in the community, often close relatives (Heyes and Frith, 2014).

## Development of the neuron-based inheritance system

The human central nervous system begins developing in the embryo around 19 days after formation of the zygote. Based on fetal movement detected by ultrasound, some functioning of the rapidly developing nervous system may be present by as early as 7 weeks of pregnancy (Marsal, 1983). In the third trimester of gestation, the developing human brain doubles in size. Much of the increase is due to a four-fold increase in cortical gray matter, with the gyri and sulci characterizing the postnatal brain being formed (Lodygensky et al., 2010). Evidence for active learning and memory functions during this period is seen by fetal responses to speech sounds of their mother's voice and native-language (Kisilevsky et al., 2009; Partanen et al., 2013). At birth, the prosodic features of a baby's cry are associated with their mother's native language (Mampe et al., 2009). In addition, newborns show left-dominant brain activation upon hearing their mother's voice compared to that of a stranger (Beauchemin et al., 2011).

Language is a prime example of acquired information passed down in a community, often by close relatives. Language influences perception and thoughts as well as serving as a primary mode for mind-to-mind communication. Neuroimaging is helping resolve a long-running debate about the role of language in perception, at least in perceiving color (Regier and Kay, 2009). Does language influence color perception or is color perception biologically-constrained by universally-shared human nervous system features? The answer derived from functional magnetic resonance imaging (fMRI) studies is that both processes are occurring in the brain, but in separate brain areas and coded in different ways (Kimbel and Delezene, 2009; Bird et al., 2014).

The human brain is about 27% of adult size at birth with virtually none of the cortical fibers myelinated, while in comparison the brain in newborn chimpanzees is 36% of adult size and neocortical myelination is around 20% of adult levels (Miller et al., 2012). Myelination of axons significantly increases the speed of electrical signals being transmitted to synaptic connections with other neurons and profoundly reduces noise from ions and other biologically active molecules in the interstitial space. Axonal myelination is critical for the full emergence of higher cognitive functions.

Full adult brain weight is reached by 7 years after birth in chimpanzees (Herndon et al., 1999) and 18–19 years in humans (Dekaban, 1978). Neocortical myelination increases to full adult levels in the chimpanzee in the motor, somatosensory and visual areas by 10–11 years of age (Miller et al., 2012). In the human, increase in neocortical myelination continues in the same regions and the prefrontal cortex until at least 28 years of age (Opris and Casanova, 2014). The neurobiology of the prefrontal cortex is of particular importance for mind-to-mind exchange of information because of its role in integrating autonomic, sensory and memory afferents from numerous brain areas for social awareness, introspection, planning, making decisions, focusing and goal-directed behavior (Bechara et al., 2000; Fleming et al., 2012). While the highest levels of synaptic density and remodeling in the prefrontal cortex are found in adolescence in humans, they only stabilize at adult levels between 30 and 40 years of age (Petanjek et al., 2011).

Although major structural features of the human brain are largely in place by the third decade of life, other processes for receiving new information and modifying informational content can continue throughout life. Most of the neurons in the human brain are present at birth. However, there is a critical exception for higher cognitive learning and memory functions. Neurogenesis continues throughout life in the hippocampus, with around 1400 new neurons generated every day (Spalding et al., 2013). The vigorous experience-dependent plasticity seen in the adult mouse cerebral cortex with new synaptic connections forming in seconds to minutes (Trachtenberg et al., 2002) provides insight into continuous active remodeling processes ongoing in human cortex. Enriching experiences induce dendritic growth and new synapses expanding cortical size, increases which can be detected in humans by MRI (Draganski et al., 2006). Dynamic remodeling of neurocircuitry in the hippocampus and the neocerebral cortex is a lifelong process in the healthy brain (Maguire et al., 2000; May et al., 2007; Anderson, 2011; Spalding et al., 2013).

## Evolutionary fitness

Evolutionary fitness is often defined simply as the capacity of an individual to survive and reproduce. As Ernst Mayr and others have emphasized, while the individual is the target for selection, evolution occurs via changes in the gene pool of populations (Mayr, 1982). Thus for gene-based heredity, the growth of populations and success in responding to environmental and ecological challenges are important metrics in assessing evolutionary fitness (Coulson et al., 2006).

Similarly, survival rate and population growth are valid measures for analyzing neuron-based evolutionary fitness. In assessing hominin evolution, a metric for analysis that makes sense is *intelligence*. Here intelligence is defined as a general mental ability that includes not only memory and learning, but also the ability to adapt to local conditions through perception, reasoning and problem solving (Gottfredson, 1997). As such, increasing intelligence in the human lineage has been a principal component for success in population growth, territorial expansion, and adapting to new environments. In a classic research paper published in 1904, the English psychologist Charles Spearman concluded “there really exists a something that we may provisionally term ‘General Sensory Discrimination’ and similarly a ‘General Intelligence,’ and further that the functional correspondence between these two is not appreciably less than absolute” (Spearman, 1904).

The *General Intelligence* (*g-factor*) described by Spearman was evidenced by a correlation between an individual's perceptive abilities in discriminating differences in light, sound and touch and life skills as estimated by verbal and mathematical abilities in school, teacher assessments and “common sense.” While Spearman's methodology was crude by modern standards, numerous studies conducted over the past century have confirmed the existence of a *g-factor* that can be estimated by psychometric tests. It is likely a measure of strongly interactive factors that include sensory and motor capabilities, number of neurons, neural network capabilities (complexity, integration, and speed), neural plasticity and cognitive capabilities such as imagination and memory.

An analysis of studies conducted to date shows a small, but highly significant correlation of ~0.2 between some aspects of general intelligence and human head size (Witelson et al., 2006; Rushton and Ankney, 2009). The correlation goes up to between 0.3 and 0.4 when mental ability is compared with brain size, depending on the cognitive modality and the study. A correlation between brain size and cognitive fitness is found in all primates, not just humans. The number of neurons increases with increasing cortical size in the primates, with neuronal number in cortical columns as much as fivefold higher compared to that in small rodents (Cahalane et al., 2014). Strong converging evidence indicates absolute brain size in many mammals is correlated with cognitive capabilities, with increasing neuronal number providing the cellular framework for organizational changes in the brain underling behavioral changes (Finlay and Uchiyama, 2015).

A correlation of 1.0 with general intelligence rather than the 0.2–0.4 that has been reported would imply that size were the sole principal component. The difference indicates the profound importance of the other factors. Indeed, the fallacy of overweighting brain size as a measure of cognitive fitness is illustrated by an outlying species dated to the terminal Pleistocene (~0.094–0.013 mya); *Homo floresiensis* had a cranial capacity similar to extant *Pan* sp. and smaller Australopithecines (~ 350 cc) and represents a noticeable departure from an otherwise consistent trend toward increasing hominin encephalization with time. Despite its small size, the brain of *H. floresiensis* is distinguished from earlier and smaller brained hominins by being morphologically derived and most similar in its organization to *H. erectus* (i.e. it possesses an expanded and derived frontal and temporal lobe and lunate sulcus position). These derived morphological states are associated with higher levels of cognitive processing than would be expected for *Pan* or *Australopithecus* (Falk et al., 2005). This presumed increase in cognitive capability is corroborated by the association of advanced and functionally diverse flaked tools with the LB1 cranium that represent a clear technological advancement from the previous Olduwan and Acheulean lithic traditions (Morwood et al., 2004).

Factors other than brain size such as organization of specialized cortical columns and domains, fast multi-modal neural networks, memory functions, imagination and plasticity exert a more significant influence on general intelligence and creative ability. But hard evidence for these factors can be only partially assessed by technological innovation in the archeological hominin record. However, estimates of brain volume are readily accessible though crania in the fossil record. In healthy adults, brain volume is on average around 8–10% smaller than the endocranial volume (Reite et al., 2010). The value of the endocranial capacity metric is it predicts probable brain size as being approximately 90% of the endocranial volume. This allows one of the factors contributing to general intelligence in the hominin line—brain size—to be traced over time. Admittedly, this is a limited metric by itself, but when coupled with functional measures such as technological achievements, an intriguing qualitative outline of increasing intelligence over time emerges in the human line (Table 3).

**Table 3:** **Endocranial Volume and Technical Achievements**.

**Evolutionary grade**	**Representative species**	**Endocranial capacity, cm^3^**	**Technology**
Australopiths (~4.4–2.0 mya)	*Australopithecus anamensis Australopithecus afarensis*, *Australopithecus garhi* *Kenyanthropus platyops* *Australopithecus africanus* *Australoputhecus sediba*	380–550^1,13^	***Lomekwian Technology***, percussion produced sharp flakes and stones.^14^
Paranthropines (~2.6–1.0 mya)	*Paranthropus aethiopicus* *Paranthropus boisei* *Paranthropus robustus*	410–550^2,13^	No archeological evidence. Presumably some use of modified non-lithic tools (i.e., sticks) and non-modified lithic tools (hammer stones).
Early Pleistocene *Homo* (~2.8–1.0 mya)	Homo habilis^*^ Homo rudolfensis^*^ Homo georgicus^*^ Homo ergaster^**^ *Homo erectus* (early) ^**^	610–1100^3,4,9,10,12^	^*^Oldowan Complex Technology^5^ Early Stone Age, percussion-induced flaking of sharp-edged stones. ^**^Acheulean complex technology Large bifacial stone tools, increasingly sophisticated flaking techniques.^6^
Late Pleistocene *Homo* (~1.0–0.3 mya)	*Homo erectus* (late) *Homo antecessor* *Homo heidelbergensis*	725–1390 ^7,11^	***Acheulean complex technology***, use of wooden spears (~0.4 mya), controlled use of fire (~ 0.79 mya), wood and rock shelters, possible mortuary practices, hafted weapons/tools.
H. neanderthalensis (~ 0.2–0.03 mya)	*Homo neanderthalensis*	1115–1835 mean = 1475 ^11^	***Mousterian complex technology*** Middle Stone Age with Increasingly sophisticated flaked tools detached from a prepared stone core.
*H. sapiens* (~ 0.19 mya – present)	*Homo sapiens sapiens* *Homo sapiens idaltu*	1205–1745 mean = 1475	Increasing evidence of complex cognitive behavior, imagination and use of symbolism.
*Pan* sp. (present)	*Pan troglodytes verus* *Pan troglodytes troglodytes* *Pan troglodytes schweinfurthii* *Pan paniscus*	300–420 ^8^	Some use of modified non-lithic tools (i.e., sticks) and non-modified lithic tools (hammer stones).

*Increasing endocranial volume, an indicator of increasing neuron-based informational capacity, closely correlates with increasingly complex technology. Evidence of the first hominins and an emerging stone tool technology are found in the Early Pleistocene. Based on the considerable morphological diversity within the cranial samples dating back to 1.8 million years ago (mya) recovered from the early Pleistocene locality of Dmanisi, Georgia, it has recently been argued that all early members of the genus Homo (H. habilis, H. rudolfensis, H. georgicus, H. erectus, H. ergaster) represent one long-lived and morphologically diverse species characterized by a trend toward reduced maxilla-facial and dental anatomy and a corresponding increase in cranial capacity (Lordkipanidze et al., 2013). Current brain size was reached around 200,000 years ago in Neanderthal and anatomically modern human populations. Neanderthals likely had more neuronal capacity dedicated to visual, somatosensory and motor functions for managing their larger bodies (Pearce et al., 2013). Smaller, gracile African hominins with modern cranial features had greater neuronal capacity to dedicate to social and cultural strategies for survival and reproduction. The endocranial volume ranges for Neanderthals and modern humans were estimated as ± 2 standard deviations of the mean. The endocranial volume of modern wild chimpanzees is shown for comparison. References: 1. (Kimbel and Delezene, 2009); 2. (Hawks, 2011); 3. (Plummer, 2004; Rightmire, 2004); 5. (Whiten et al., 2009); 6. (Ambrose, 2001); 7. (Pearce et al., 2013); 8. (Zihlman et al., 2008); 9. (Spoor et al., 2015); 10. (Villmoare et al., 2015); 11. (Smith, 2002); 12. (Dunsworth and Walker, 2002); 13. (White, 2002); 14. (Harmand et al., 2015)*.

## Bipartite evolution: genetic changes enhancing neuron-based heredity

### Language

***One*** of the major differences between the human brain and that of nonhuman primates, including chimpanzees, is found in the left cerebral cortex. A large crescent-shaped region encompassing components of the temporal, occipital, parietal, and frontal lobes has specialized high-functioning neural assemblages for observational and communicative skills. In the caudal region of the crescent where the temporal, parietal, and occipital lobe converge lies Wernicke's area, a crucial site for language comprehension (enhanced observational skills). Anchoring the rostral region of the crescent is Broca's area in the frontal cortex, crucial for spoken language (advanced communicative skills).

There are a number of asymmetrical features of the human left cerebral cortex not found in chimpanzees and other nonhuman primates (Chance, 2014) indicative of important genetic changes in the hominin lineage. Human-specific features include larger pyramidal neurons in the left cortex compared to the right in specialized language areas and some specialized visual processing areas (Hutsler, 2003). Pyramidal neurons and interneurons in the neocortex are organized in linear radial arrays called minicolumns (Mountcastle, 1997). Neurons in a minicolumn share common response features and electrical activity can be amplified through increasing the number of neuronal processes and synaptic connections in the adjacent neuropil space. Minicolumns in the left human planum temporale, a triangular section of the superior temporal lobe in Wernicke' area, are larger in the left cortex than on the right side (Buxhoeveden et al., 2001). Another important difference is that axons in the left human posterior superior temporal lobe have thicker myelin sheaths providing more precise, faster electrical signal conductance in their neural assemblages (Anderson et al., 1999).

While it is still not known why language capabilities are concentrated in the left hemisphere, progress has been made in identifying the critical mutations involved. A single nucleotide mutation in the FOXP2 gene was found in 2001 to be responsible for severe speech and language dysfunctions in some members of a large English family (Watkins et al., 2002a). The pattern of inheritance is classical autosomal dominance. Family members carrying the mutant gene are afflicted and their children have a 50% chance for inheriting the genetic dysfunction. The discovery focused scientific attention on learning more about the previously obscure gene and the protein it encoded.

Affected family members have pronounced cognitive and motor deficits. Speech problems manifest early in childhood and persist throughout life. Ungrammatical word order and confused endings make it difficult or impossible to determine what they are saying. Affected individuals also score lower on non-verbal intelligence tests than non-affected family members.

Some developmental deficits from the mutant gene are evident in high-resolution MRI brain scans (Watkins et al., 2002b). There is less neuron-rich gray matter bilaterally in areas of the frontal cortex, somatosensory cortex, temporal lobe and an approximate 20% loss of neuron-rich tissue in the caudate nucleus. The frontal lobe is a higher associative cortical area intimately engaged in cognitive processes. The caudate nucleus in the basal ganglia has a major role in selection and control of language use, coordinating motor output with the putamen (Hervais-Adelman et al., 2014).

The behavioral and structural deficits are consistent with molecular studies showing FOXP2 protein binds to number of gene targets in the developing human frontal cortex and basal ganglia (Spiteri et al., 2007). In these studies, FOXP2 was shown to function as a transcription factor meaning that it regulated expression of many other genes like a conductor leading a symphony orchestra. Collectively, evidence from molecular, cellular, animal and human studies strongly support a crucial role for FOXP2 in orchestrating the development and neuroplasticity of neural circuitry underlying motor learning of language functions (Preuss, 2012).

The FOXP2 protein differs by just 2 out of 714 amino acids between chimpanzees and humans (Enard et al., 2002). But these differences may be profoundly magnified because the protein regulates the expression of numerous genes during development. The FOXP2 gene sequence in modern humans has been found in the Neanderthal genome, dating its existence in the human lineage back to the last common ancestor shared by these sister species. However, changes in regulatory elements between Neanderthals and modern humans may have increased the efficiency of the gene's transcription in the present day population (Maricic et al., 2013).

### Goal-directed and social behavior

The prefrontal cortex accounts for one-third of the human cerebrum. It constitutes the largest region of the frontal lobe; the other areas being the motor and premotor cortices. The central role played by the prefrontal cortex in the evolution of modern humans is evident in its functions: introspection, goal selection, planning, decisiveness, social awareness and social behavior (Damasio et al., 1994; Eslinger et al., 2004; Fleming et al., 2012). Major differences have been identified between the human and chimpanzee prefrontal cortex. Dendrites of human pyramidal neurons are significantly longer and have more branches (Bianchi et al., 2013). The number of axons, dendrites and synapses is significantly higher (Semendeferi et al., 2011; Spocter et al., 2012). Consistent with the concept of a major increase in connectivity and neural network complexity in the human lineage, the human prefrontal cortex is *in toto* allometrically larger and asymmetrically larger in the left hemisphere with a greater volume of axon-rich white matter to neuron-rich gray matter than in chimpanzees and other nonhuman primates (Smaers et al., 2011; Passingham and Smaers, 2014).

An indication of the importance of the prefrontal cortex in human evolution is shown by metabolomic studies demonstrating this region of the brain has genetically evolved at a four-fold greater rate than predicted in differing from chimpanzees (Bozek et al., 2014). At the same time, the human skeletal muscle metabolome has changed at a seven-fold or higher rate from the chimpanzee. This reflects a major shift in energy resources from muscle use to a critical brain area and is consistent with the known enhanced cognitive functions of the human prefrontal cortex and the greater muscular strength of chimpanzees.

### Genetic regulation of brain size

Some genes associated with developmental disorders causing microcephaly have been identified. In individuals with head sizes three standard deviations and more below the mean, slightly over 50% had an IQ lower than 70 and none had an IQ above 100 (Dolk, 1991). Seven genes have now been identified for microcephaly (Gilmore and Walsh, 2013). All mutations are associated with cellular centrosomal functions indicating the mutated genes affect mitosis in the developing nervous system. Based on this and other cellular centrosome functions, the wild type of the microcephaly genes would be predicted to contribute to normal brain development.

There is evidence wild type microcephalin and ASPM, the two genes in which loss-of-function mutations account for most cases of primary microcephaly, have been important in the evolution of larger brains in the primate lineage (Evans et al., 2004; Ali and Meier, 2008). Significant progress has been made in understanding the molecular mechanisms underlying the severe reduction in the number of neurons and size of the cerebral cortex resulting from mutations of ASPM. Wild type ASPM regulates Wnt signaling activity (Buchman et al., 2011), which promotes neurogenesis and neuronal development, in part through the FZD8 receptor. Mutations of ASPM can lead to a dramatic reduction in cortical size. The importance of the Wnt-FZD8 pathway has been recently highlighted by the demonstration of a human-accelerated regulatory enhancer (HARE5) functionally linked with FZD8 (Boyd et al., 2015). Human HARE5 differs by 16 out of 1219 base pairs from the chimpanzee HARE5 locus and in comparative studies promotes larger increases of neuronal progenitor cells in the transgenic mouse cortex (Boyd et al., 2015).

Despite large genome-wide screening studies, single genes correlated with large effects on brain size and intelligence other than those discussed in the preceding paragraphs have not been identified (Butcher et al., 2008; Chabris et al., 2012). Rather as Plomin and his colleagues have emphasized, the genetic heredity of complex traits like intelligence is largely due to interactions between many genes, each exerting a small effect (Plomin et al., 2009). However and most importantly, while an individual's genome remains constant over their lifetime, genetic influences on complex traits can change with age. For intelligence, meta-analysis of results from twin studies indicate genetic heritability increases from 20% in infancy to 40% in childhood and up to 60% or more in adulthood (McClearn et al., 1997; Plomin and Deary, 2015). Thus while the current intense scientific focus on genetic factors contributing to intelligence is justified, these data demonstrate that other significant factors are at work and should also be investigated.

## The great brain race

**Figure 1:**
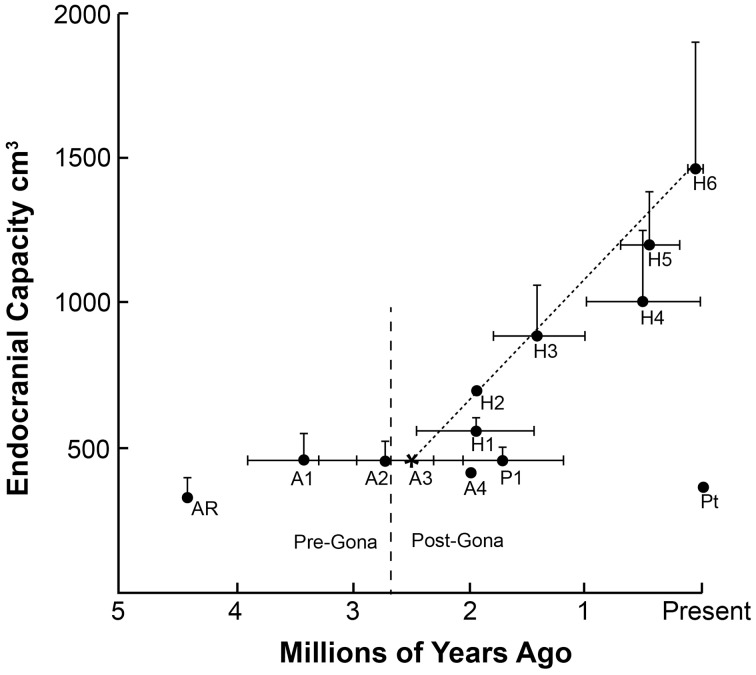
**The chronology of appearance and extinction of major species in the hominin lineage are shown**. In the big picture, the period in which stone tools were being manufactured at Gona 2.6 million years ago coincides with a time of great change in Australopithecine lines, perhaps in response to increasing seasonality and climatic fluctuations. Both the gracile, larger-brain members of the genus *Homo* and the cranio-dentally robust Paranthropines appear at this time. The mean and largest known endocranial volume for each species are plotted to illustrate the almost linear increase in brain size in the *Homo* genus over the past 2.5 million years. As noted earlier, recently it has been argued that all early members of the genus *Homo* (e.g., *H. habilis, H. rudolfensis, H. erectus*) represent one long-lived and morphologically diverse species (see Lordkipanidze et al., 2013). The endocranial capacity of modern chimpanzees *Pan troglodytes* (Pt) is included for comparison. Ar (*Ardipithecus ramidus)*; A1 (*Australopithecus afarensis)*; A2 (*Australopithecus africanus)*; A3 (*Australopithecus garhi)*; A4 (*Australopithecus sediba*); P1 (*Paranthropus boisei)*; H1 (*Homo habilis)*; H2 (*Homo rudolfensis)*; H3 (*Homo erectus* [early]); H4 (*Homo erectus* [late]); H5 (*Homo heidelbergensis)*; H6 (*Homo sapiens)*. Sources: (Falk et al., 2000; Plummer, 2004; Rightmire, 2004; Zihlman et al., 2008; Kimbel and Delezene, 2009; Suwa et al., 2009; Hawks, 2011; Pearce et al., 2013).

There must have been a tipping point in hominin evolution where neuron-based brain-centered processes had become so critical for the survival of communities that they began setting the genetic agenda. Charting the beginning of increased technological capabilities and dramatic increases in brain size as indicators of increasing general intelligence, this point was reached sometime between 3.3 and 2.6 mya (Figure 1). This range includes the appearance of the first hominin fossils 2.8 mya (Villmoare et al., 2015) and is based on the oldest unequivocal examples of modified stones tools found in Lomekwi, West Turkana, Kenya dating back 3.3 mya (Harmand et al., 2015) and in the Gona River drainage area of the Awash Valley in Ethiopia in which radioisotope dating indicates were knapped 2.6 mya (Semaw et al., 1997).

From a neuroscience perspective, the importance of the knapped stones in Lomekwi and Gona is what they reveal about the cognitive and motor capabilities of the toolmakers. First, production of tools using replicative techniques indicates the *general intelligence* level in these communities was beginning to exceed that of wild chimpanzees. Manufacturing the stone tools was likely just part of a complex set of community behaviors involved in procuring and processing available food resources for consumption. Like chimpanzees, hominins in Lomekwi and Gona were probably omnivores and may have acquired meat through opportunistic scavenging, an activity that is both dangerous and would require a coordinated group effort given the potential competition for carrion with non-hominin scavengers.

Butchering animals is a skill and likely a community activity. Either the tools needed to be carried to the carcass or vice-versa. Producing the tools required perceptive and planning skills for collecting the right type of stones to be knapped and then motor skills using a deliberate set of motor movements to produce conchoidal fractures (fractures with smooth shell-like convexities and concavities) to produce sharp-edged flakes and larger tools with sharp edges (Ambrose, 2001). The cognitive functions for acquiring and using information for conducting this series of sequential actions are indicative of enhanced learning of motor skills along with planning and memory capabilities beginning to edge beyond those of extant wild chimpanzees. Chimpanzees do use stone hammers to crack nuts and in the process incidentally produce some stone flakes (Mercader et al., 2002). The flakes accumulate in repeatedly used sites. It remains controversial on how much resemblance the chimpanzee flake assemblages have with ancient tool making sites like Gona (Vogel, 2002).

**Figure 2:**
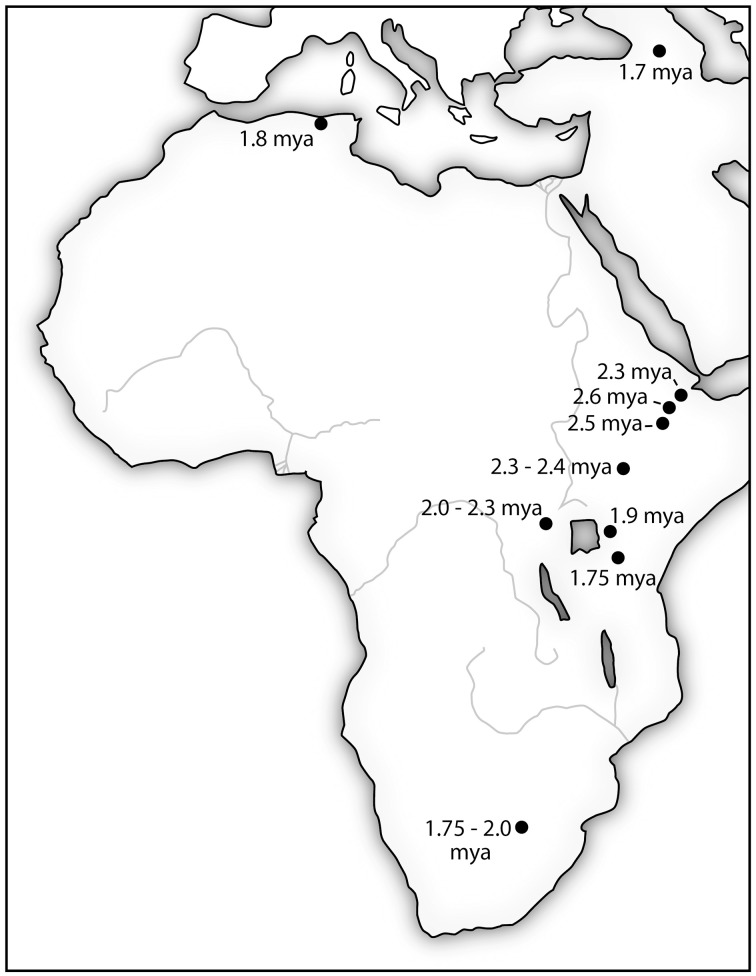
**The location of stone tools manufactured using Oldowan Complex technology (•) are initially clustered in the East African Rift Valley**. The expansion of the technology over nearly a million-year period coincides with increasing brain size in hominins. It is also consistent with an increasing general intelligence as indicated by adaptability to new environments. Sources: (Plummer, 2004; Schick and Toth, 2006; Semaw, 2006).

What is so critically important about Lomekwi and Gona is that they provide hard evidence for a viable idea being created, transmitted and replicated from mind-to-mind in hominins. Producing stone tools for cutting and other general purposes was likely only part of what was going on in hominin communities 3.3–2.6 mya. Meeting the challenges posed by climatic fluctuation and increasing seasonality, complex social coalitions and inter- and intraspecific competition for food resources were likely co-factors driving hominin evolution. In the stiff competition for resources, it was not only the intelligence of single individuals that was advantageous, but also the intelligence level of the community. Having individual hominins who realized the value of tools and had the ability to make and use them was one major advance; the other was the ability to successfully transmit this knowledge to other members of the community. Other members of the community had to possess a sufficient *general intelligence* to “get it.” The continuity of the Oldowan technology from 2.6 mya for the next million years spreading to an ever widening swath of sites across Africa and into Eurasia shows that the Gona hominins and hominins that followed “got it” and were able to pass important information for survival from generation to generation (see Figure 2). Currently, there is insufficient evidence in the archeological record to determine if the earlier tool manufacturing skills demonstrated by Lomekwian hominins were successfully transmitted to following generations.

Brain size is the outward manifestation of the high premium placed on new mutations and genetic combinations which further increased the *general intelligence* of the community. Communities with the higher collective intelligence levels were those that most quickly understood and adapted to local conditions, the “fittest” with the greatest likelihood for survival. Groups with the technology would have a significant advantage over less technologically-adept competitors.

Although the hominin fossil record preserves evidence of a diverse adaptive radiation with numerous genera and species, many of which were contemporaries from differing geographical locations in East and South Africa, there is little evidence of hominin sympatry prior to the beginning of the Pleistocene (~2.5 mya). Beginning ~ 2.0 mya, however, there is fossil evidence of both the geological and temporal overlap between multiple hominin species representing the genus *Paranthropus* (*P. boisei, P. robustus*) and the genus *Homo* (*H. habilis, H. rudolfensis, H. ergaster*) at Olduvai Gorge (Tanzania) Koobi Fora (East Rudolf, Kenya) and Swartkrans (South Africa) (Wood, 1991; Schwartz and Tattersall, 2003a,b; Gathogo and Brown, 2006). While the fossil record can only demonstrate that these taxa are contemporaneous and that they occur within the same stratigraphic levels associated with specific temporal and geographical points in the distant past, the evidence from Olduvai, Koobi Fora and Swartkrans is consistent with the interpretation of a paleolandscape simultaneously occupied by multiple hominin taxa. All were likely to have been ecological generalists that would have experienced at least modest levels of direct competition with other hominin taxa (Wood and Strait, 2004). If the early Pleistocene hominin community was indeed characterized by widespread sympatry then mind-to-mind transfer of cultural and technological information would have been a critical mechanism for survival and out-competing interspecific contemporaries.

In the 800,000 year period following the advent of the new technology at Gona, fossils from early Pleistocene members of the genus Homo provide tangible evidence of rapidly increasing encephalization associated with increasingly refined and advanced lithic tool making traditions and a paleolandscape occupied by multiple hominin taxa. They were winning *The Great Brain Race*, significantly advancing the Oldowan technology. Members of hominin communities developed better knapping techniques for larger, more sophisticated cutting tools - the Acheulean Industry (Plummer, 2004; Lepre et al., 2011). With brain size increasing over time to roughly twice the size as the typical Australopithecine, early hominins were remarkably fit and versatile. Bands of these adaptable hominins roamed over much of Africa and Asia from 1.8 mya until as recently as 0.03 mya. Their ability to thrive in many diverse environments suggests enhanced intelligence and neural plasticity as well as increased brain size.

*The Great Brain Race* continued with increasing brain size in later late Pleistocene hominin communities with new emerging species including *H. antecessor* and *H. heidelbergensis*. By 400,000 years ago, cranial capacity in *H. heidelbergensis* was reaching volumes in the modern range. Anatomically modern humans and Neanderthals are sister taxa thought to be descendants from a common ancestor (most often identified as *H. heidelbergensis* or *H. antecessor*), but the cranial fossil record is fragmentary. The technological record is clearer. Preceding the advent of modern humans, the Middle Stone Age gradually began on the East Coast of Africa around 250,000 to 300,000 years ago, with pronounced advances in stone tool manufacturing technique (McBrearty and Brooks, 2000).

The first evidence of anatomically modern humans consists of fossilized skulls with modern human features unearthed in southern Ethiopia that date back to 190,000–200,000 years ago (McDougall et al., 2008). Molecular dating techniques indicate a similar time frame. Ochre (iron oxide rich clay) processing tools and engraved pieces dating back 100,000 years ago have been discovered in Blombos Cave in South Africa suggesting the manufacturers possessed complex cognitive skills, including imagination and the ability to use symbolism for encoding information (Henshilwood et al., 2011).

The skill of Stone Age artisans in the past 100,000 years is also shown in their manufacture of composite tools such as sharp-edged knapped stones mounted in wooden handles (hafts) producing, for example, axes, knives, and spears. Recent re-creations of the hafting processes used by craftsmen working in the Sibudu Cave, South Africa over 70,000 years ago suggest those individuals possessed the essential elements of the modern mind: excellent working memory and intelligence in reasoning, planning, and comprehending complex ideas (Wadley et al., 2009; Wynn, 2009).

Populations of anatomically modern humans had begun migrating out of northern Africa into southern Asia 50,000–60,000 years ago (Mellars et al., 2013). As the bands spread out into Eurasia, they encountered populations of Neanderthals and Denisovans. Genetic studies indicate interbreeding between anatomically modern humans and Neanderthals before the latter disappeared from the fossil record around 30,000 years ago. While estimates vary, some studies suggest from 3 to 7% of the non-African modern human genome comes from Neanderthal ancestry (Wall et al., 2013; Lohse and Frantz, 2014). Similarly, 4–6% of the genome of Melanesians and Australian Abrogines is derived from a Denisovan population (Krause et al., 2010; Rasmussen et al., 2011; Reich et al., 2011). It can be posited that human evolution since these admixtures into the gene pool has been largely through genetic refinement (selection of existing genes providing advantages for survival and reproduction) and neuron-based inheritance of acquired information.

### Neuron-based evolution: neolamarckian features

Like many issues in cultural heredity, the nature of the evolutionary processes involved is controversial. Blackmore (1999) posits it is Lamarckian; Jablonka and Lamb (2007) agree that there are Lamarckian aspects to cultural evolution, while Richerson et al. (2010) use the term gene-culture coevolution. The concern with the term “cultural evolution” is it refers to phenomena, not the underlying biological processes. The three principal components of cultural evolution are gene-based and neuron-based systems interacting with environmental influences. The challenge is to determine the contribution of each of these three factors to an observed cultural feature.

Where there is consensus is that the history of human evolution since the advent of Oldowan Technology is closely associated with the generation of new technologies, culture developments and ideas which are transferred from mind-to-mind, Many of the neuron-based mechanisms in this process are seen in hippocampal functions. The role of the hippocampus in encoding new memories is critical in neuron-based heredity. The generation of new neurons continues throughout life in the human hippocampus with approximately 1400 new nerve cells added each day (Spalding et al., 2013). The new neurons are important in the dynamic hippocampal process of organizing modules in the cortex for storing memories (Frankland and Bontempi, 2006). Neural activity strengthening relevant synaptic connections and establishing new synaptic neural circuitry are essential components for new memories (Anderson, 2011).

As discussed earlier, mind-to-mind transfer begins before birth with fetal brain activation seen in response to their mother's voice and native language. Vocabulary changes throughout life with use and disuse of words. For example, bilinguals experience a loss in the vocabulary of their first language when immersed in an environment that predominantly uses their second language (Goral et al., 2008). Other examples of remodeling of neural circuitry throughout life are seen in hippocampal size and function. Activities stimulating hippocampal activity (use) increase hippocampal size and can improve memory. The classical example is the larger posterior hippocampus of highly trained taxi drivers in London with size significantly correlated with months of professional driving (Maguire et al., 2000). Significant increase in hippocampal size has also been shown in German students spending 3 months of intensive study preparing for an entrance exam into medical school requiring a high level of information encoding, retrieval and use (Draganski et al., 2006). Hippocampal atrophy of 1–2% a year occurs in normal aging, but can be reversed with regular aerobic exercise. In older individuals in their 60 and 70s, regular exercise for a year led to an average increase in hippocampal size of 2% and improved memory processes (Erickson et al., 2011).

The intense focus on developing and maintaining navigational skills by London taxi drivers, while significantly increasing spatial skills and structural size of the posterior hippocampus, is associated with decreased structural size of the anterior hippocampus and decreased associative memory functions compared to controls (Woollett and Maguire, 2009). Again as discussed earlier, intense “Use” and “Disuse” as mechanisms engaged in intentionally meeting new needs and leading to new modes of living, habits and functions are consist with Neolamarckian concepts of heredity (Packard, 1901).

Given the advanced state of genetics research, detailed knowledge of molecular pathways and mechanisms of action as well as readily available sophisticated methodology for scientific studies, how can knowledge of the second system of heredity significantly add to our understanding of human biology? Why is it important? As numerous studies have repeatedly shown, complex traits including autism, attention deficit/hyperactivity disorder and general intelligence are not 100% genetically heritable (Ronald and Hoekstra, 2011; Franke et al., 2012; Plomin and Deary, 2015). As discussed earlier, while the genome is virtually stable over an individual's lifespan, the genetic heritability of intelligence changes from 20% at infancy to greater than 60% in adulthood. Other interactive factors are at work altering the balance between genes, the neural system and the environment. Increasing awareness of neuron-based heredity as a system adds another tool to use in analyses of factors contributing to complex behavior traits, providing insights that could lead to the development of new treatments for common neurological disorders.

For other overlapping disciplines, it is equally important. The fossil and archeological record of human origins is characterized by an ever-increasing reliance on cultural and technological innovation. Initially the pace and tempo of human evolution is primarily influenced by genetic mechanisms of heredity. However, the rapid pace of technological innovation and advancements in material culture typical of the early Pleistocene, and most often associated with the origins of the genus *Homo*, demonstrate that other, most likely neuronal-based, mechanisms must be at work and are now significantly influencing the human lineage. Consequently, when biological anthropologists interpret and contextualize the human fossil and archeological records to answer questions about why some species were successful, why some went extinct and how earlier hominins may be related to later more derived hominins, it is critical that these interpretations include considerations of both genetic and neuronal hereditary mechanisms. Only through an increased understanding of the separate yet complementary influences of genetic and neuronal hereditary will it be possible to truly decipher the complexities of human evolution and the nexus of biology, culture and technology that defines our lineage.

Advances in neuroscience now make it possible to study structural and functional processes in the brain associated with acquiring cultural and behavioral information and transmission from mind-to-mind. One of the exciting challenges will be to see what new insights are gained into the descent of anatomically modern humans from a common ancestor shared with great apes. As Darwin explained in *The Origin of Species*, “No one should be surprised at much remaining as yet unexplained in regard to the origin of species and varieties” (Darwin, 1859). Much progress has been made since then in understanding genetic inheritance and its contributions to evolution. But much remains to be learned about neuron-based heredity, including its extraordinarily important role in hominin evolution.

## Author contributions

DMG is a neuroscientist who has published over 200 papers and reviews, primarily on neurodegenerative diseases and aging of the brain. He developed the hypothesis and substantially contributed to the analysis and interpretation of this work. ASD is a paleoanthropologist who has published on ape and hominin evolution. He made substantial contributions to the analysis and interpretation of this study.

### Conflict of interest statement

The authors declare that the research was conducted in the absence of any commercial or financial relationships that could be construed as a potential conflict of interest.
